# A data‐driven approach to complement the A/T/(N) classification system using CSF biomarkers

**DOI:** 10.1111/cns.14382

**Published:** 2023-07-27

**Authors:** Laura Hernández‐Lorenzo, Maria José Gil‐Moreno, Isabel Ortega‐Madueño, Maria Cruz Cárdenas, Maria Diez‐Cirarda, Alfonso Delgado‐Álvarez, Marta Palacios‐Sarmiento, Jorge Matias‐Guiu, Silvia Corrochano, José L. Ayala, Jordi A. Matias‐Guiu, for the Alzheimer's Disease Neuroimaging Initiative

**Affiliations:** ^1^ Department of Neurology San Carlos Research Institute (IdSSC), Hospital Clínico San Carlos Madrid Spain; ^2^ Department of Computer Architecture and Automation, Computer Science Faculty Complutense University of Madrid Madrid Spain; ^3^ Department of Clinical Analysis, Institute of Laboratory Medicine IdSSC, Hospital Clínico San Carlos Madrid Spain

**Keywords:** Alzheimer's disease, cerebrospinal fluid, clustering analysis, early detection, machine learning, mild cognitive impairment

## Abstract

**Aims:**

The AT(N) classification system not only improved the biological characterization of Alzheimer's disease (AD) but also raised challenges for its clinical application. Unbiased, data‐driven techniques such as clustering may help optimize it, rendering informative categories on biomarkers' values.

**Methods:**

We compared the diagnostic and prognostic abilities of CSF biomarkers clustering results against their AT(N) classification. We studied clinical (patients from our center) and research (Alzheimer's Disease Neuroimaging Initiative) cohorts. The studied CSF biomarkers included Aβ(1–42), Aβ(1–42)/Aβ(1–40) ratio, tTau, and pTau.

**Results:**

The optimal solution yielded three clusters in both cohorts, significantly different in diagnosis, AT(N) classification, values distribution, and survival. We defined these three CSF groups as (i) non‐defined or unrelated to AD, (ii) early stages and/or more delayed risk of conversion to dementia, and (iii) more severe cognitive impairment subjects with faster progression to dementia.

**Conclusion:**

We propose this data‐driven three‐group classification as a meaningful and straightforward approach to evaluating the risk of conversion to dementia, complementary to the AT(N) system classification.

## Graphical abstract

We compared CSF biomarkers clustering results against their AT(N) classification in two cohorts. Clustering yielded three groups: non‐defined AD, early stages with delayed risk of dementia, and severe cognitive impairment subjects with faster progression to dementia. We propose this data‐driven categorization as a meaningful and straightforward approach to evaluating dementia risk, complementary to the AT(N) system classification.

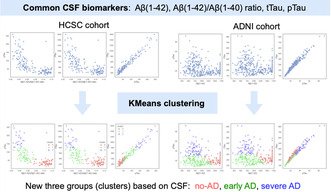

## 1 INTRODUCTION

Alzheimer's disease (AD) is the most common cause of dementia.^1^ Clinical and epidemiological evidence indicates that AD‐related pathological changes occur decades before the onset of clinical symptoms.^2^ Identifying patients at risk in stages such as mild cognitive impairment (MCI) is essential to provide a critical time window for early clinical management, treatment, care planning, and design of clinical trials.^3^, ^4^ Currently, early and differential AD diagnosis is performed by checking different biomarkers related to biological processes underlying the disease, such as β‐amyloid accumulation and neurofibrillary tangles formation.

Different modalities of AD biomarkers have been implemented in the clinical characterization of MCI patients, combining neuropsychological tests and molecular and neuroimaging biomarkers.^5^, ^6^, ^7^ Advances in the study of biomarkers have enhanced a redefinition of Alzheimer's disease (AD) as a biological construct, meaning different pathological pathways and systemic processes involved. In 2016, Jack et al.^6^ proposed a new classification scheme based on biomarkers for three dimensions: AT(N). Under this definition, the AT(N) classification system recognizes three general groups of biomarkers for AD: biomarkers of β‐amyloid plaques (A), fibrillar tau (T), and neurodegeneration or neuronal injury (N).^6^ Amyloid positron emission tomography (PET); cerebrospinal fluid (CSF) Aβ1–42 and Aβ1–42/Aβ1–40 ratio correspond to the “A” category; tau‐PET‐ and CSF‐phosphorylated Tau (pTau) correspond to the “T” category; and ^18^F‐fluorodeoxyglucose‐PET (FDG‐PET), structural brain MRI, and CSF total Tau (tTau) are considered markers of the “N” category. Thus, the AT(N) classification scheme is presented as an unbiased descriptive system that can be applied to all patients without suspected AD cognitive symptoms.

Despite its wide use in recent years, some studies have raised issues associated with the AT(N) classification system. One study^8^ showed that the AT(N) system was inconsistent and highly dependent on the biomarkers used and the stages of the disease, even when using several cut‐off points. This work also exhibited a very low correlation between the distinct biomarkers in the whole sample set and the AD continuum stages, except for CSF pTau and tTau.^8^ In addition, another work^9^ found a large number of profiles according to the AT(N) system in their application to a memory clinic, but with an important overlap in baseline characteristics and patterns of cognitive decline.^9^

There are still a few challenges in applying the AT(N) system to the clinic. We hypothesized that applying unbiased, data‐driven techniques such as machine learning (ML) algorithms might help optimize it. This approach has gained a particular interest in medicine, supporting diagnosis, prognosis, and treatment.^10^, ^11^, ^12^ Within ML methods, a large group is known as “unsupervised classification” techniques, which includes clustering algorithms. These algorithms can find patterns within the data to identify new groups or clusters. Although unsupervised classification techniques are less common than supervised classification (prediction of output from input data), clustering applications in a clinical context could discover new subgroups or types of patients. The evaluation of newly discovered clusters offers new knowledge about how the disease occurs, especially when applied in the complex context of highly heterogeneous diseases such as AD.^13^ These clustering techniques can be applied to obtain categories based on the values of different biomarkers, similar to what is done by the AT(N) system.

One of the main goals of the AT(N) classification system is to get insight into the evolution of the biomarkers in AD and eventually be able to make an early (even preclinical) biomarker‐based diagnosis. Accordingly, we set a very similar goal from the point of view of a data‐driven strategy such as clustering. In this work, we present a clustering analysis of the CSF biomarkers representative of the AT(N) system: Aβ1–42, Aβ1–42/Aβ1–40 ratio, pTau, and tTau. The main objective of our study was to compare the clustering results over all available CSF biomarkers against the AT(N) system. Therefore, we evaluated the diagnostic and prognostic capacity of the clusters and interpreted their significance from the point of view of AD progression. Finally, we validated our results in two cohorts of AD patients: the Alzheimer's Disease Neuroimaging Initiative (ADNI) and data from our center, representing both research and clinical practice settings, respectively.

## 2 MATERIALS AND METHODS

### 2.1 Datasets description

We used two data sources: (i) data coming from the San Carlos Clinical Hospital in Madrid, Spain (named HCSC dataset throughout this work), and (ii) data coming from the Alzheimer's Disease Neuroimaging Initiative (ADNI) (adni.loni.usc.edu) database. In ADNI and HCSC data, we queried for cerebrospinal fluid (CSF) biomarkers measurements, sociodemographic information, and clinical diagnoses. CSF measurements included Aβ(1–42) (raw value and ratio with Aβ(1–40); this last one only in the case of the HCSC dataset), phospho‐tau (pTau), and total tau (tTau). Table 1 shows the main demographic and clinical characteristics of the HCSC and ADNI datasets.

**TABLE 1:** Sample description of the HCSC and ADNI datasets.

Dataset	Variable	LMCI	EMCI	SMC	MCI‐NN	CN
HCSC	Number	63	26	43	33	NA
	Sex, Female (%)	33 (53.12%)	16 (54.55%)	22 (51.16%)	18 (54.55%)	NA
	Age (years) at baseline	74.98 ± 4.97	74.19 ± 6.11	65.93 ± 9.56	66.52 ± 10.16	NA
	Education years	10.46 ± 4.42	9.04 ± 5.39	12.08 ± 4.39	12.10 ± 5.44	NA
	MMSE at baseline	24.26 ± 3.57	26.29 ± 2.59	28.03 ± 1.97	26.88 ± 2.33	NA
ADNI	Number	99	111	50	NA	159
	Sex, Female (%)	38 (38.38%)	47 (42.34%)	30 (60.00%)	NA	79 (49.69%)
	Age (years) at baseline	72.37 ± 7.54	71.59 ± 6.97	71.09 ± 5.00	NA	73.97 ± 5.86
	Education years	16.28 ± 2.81	15.91 ± 2.68	16.46 ± 2.62	NA	16.29 ± 2.76
	MMSE at baseline	27.52 ± 1.72	28.21 ± 1.63	29.04 ± 1.19	NA	29.05 ± 1.13

Abbreviations: CN, Cognitively Normal; EMCI, Early Mild Cognitive Impairment due to AD; LMCI, Late Mild Cognitive Impairment; MCI‐NN, MCI without evidence of neurodegeneration; MMSE, Mini‐Mental State Examination; SMC, Subjective Memory Complaints.

The HCSC cohort data included 165 patients consulting for memory loss recruited from the Department of Neurology between December 2018 and December 2021. All patients were Spaniards and were native Spanish speakers. All patients were examined with a comprehensive neuropsychological protocol, structural neuroimage (TC or MRI), and lumbar puncture for CSF biomarkers. Patients were categorized, according to the results from the neuropsychological assessment, longitudinal follow‐up, and results from cerebrospinal fluid biomarkers, as follows: subjective memory complaints (SMC) (*n* = 43), early mild cognitive impairment due to AD (EMCI) (*n* = 26), and late MCI due to AD (LMCI) (*n* = 63) and MCI without evidence of neurodegeneration (MCI‐NN) (*n* = 33). Suspected non‐Alzheimer's neurodegenerative disease patients (frontotemporal dementia and Lewy body disease) were excluded. Patients were considered AD when the diagnosis was supported by altered levels of Aβ(1–42) or altered Aβ(1–42)/Aβ(1–40) ratio and pTau in CSF analysis according to diagnostic criteria.^14^ For more information about the neuropsychological protocol, CSF samples acquisition and analysis, and diagnostic criteria employed in the HCSC dataset, please refer to Appendix S1: Supplementary Information 1.1 and 1.2. Diagnostic criteria are depicted in Table S1.

The ADNI database was launched in 2003 as a public–private partnership, led by Principal Investigator Michael W. Weiner, MD, and has as its primary goal to test the combination of several neuroimaging techniques, biological markers, and clinical and neuropsychological evaluations to assess the progression of mild cognitive impairment (MCI) and early AD. We obtained data from the key table “ADNIMERGE,” which contains a summary of essential features and measurements of subjects enrolled in ADNI distributed in medical visits. We selected only patients from the ADNIMERGE table whose diagnosis was available for 5 years or more (*n* = 625). Next, we discarded patients who reversed diagnosis during the follow‐up (e.g., MCI to CN or dementia to MCI) or started with a dementia diagnosis (*n* = 581). Lastly, we selected patients with available CSF biomarkers measurements at the baseline visit (*t =* 0). As a result, 419 patients remained for this analysis. Patients were categorized into cognitively normal (CN) (*n* = 159), subjective memory complaints (SMC) (*n* = 50), early mild cognitive impairment (EMCI) (*n* = 111), and late MCI (LMCI) (*n* = 99). Please refer to Table S2 for ADNI diagnosis criteria.

### 2.2 Classification according to the AT(N) system

Subjects in both datasets were categorized according to AT(N) system.^6^ A+ was considered when the value of Aβ42 and/or Aβ42/Aβ40 was lower than reference values, and T+ and N+ when pTau and tTau were higher, respectively. We used reference values of 0.068 (Aβ(1–42)/Aβ(1–40) ratio), 723 pg/mL (Aβ(1–42) if the ratio was not available), 59 pg/mL (pTau), and 410 pg/mL (tTau) in HCSC dataset,^15^ and cut‐off points of 980 pg/mL (Aβ(1–42)), 24 pg/mL (pTau), and 266 pg/mL (tTau) for ADNI.^16^, ^17^

### 2.3 Statistical analysis

#### 2.3.1 Proposed generalization analysis

We trained two different KMeans models for HCSC and ADNI cohorts because the latter did not include the amyloid ratio present in HCSC. However, to properly generalize the three clusters obtained and discussed in this work regarding CSF biomarkers, we evaluated the clustering results using another smaller cohort from ADNI that included data of the Aβ(1–42)/Aβ(1–40) ratio. For selected patients in this ADNI cohort, we followed the steps described in the first section of Materials and Methods. We employed the HCSC cohort as the discovery cohort (*n* = 165) and the mentioned ADNI cohort as the validation cohort (*n* = 174). Next, we performed the following steps. First, with the trained clustering model on the discovery cohort, we labeled the discovery cohort according to cluster assignments. Secondly, we trained a logistic regression model on biomarkers values in the discovery cohort, using cluster assignations as the classification labels. Finally, we evaluated this classification model on the discovery cohort, obtaining classification performance metrics such as accuracy, the area under the ROC curve (AUC), and Matthews correlation coefficient (MCC).

#### 2.3.2 CSF biomarkers clustering

We performed a clustering analysis through KMeans with Euclidean distance. For this, we used as input all the available biomarkers measurements combined and obtained results in the HCSC and ADNI datasets. The biomarkers values were scaled to the unit standard deviation. We implemented standardization and KMeans analyses using Scikit‐Learn v.1.1.2.^18^ KMeans algorithm needs the number of clusters as input. This number was set from 2 to 10 clusters to evaluate the quality of different clustering solutions using the silhouette index (SI) score. This score is based on the intra‐ and inter‐cluster distance, that is, the cohesion and separation of clusters, respectively. This value ranges from 0 to 1, where 1 represents a perfectly clustered dataset. Moreover, we described each cluster's diagnosis and AT(N) categories distributions. We also described the clinical characteristics of all clusters and compared them using ANOVA, considering significant *p*‐values < 0.05.

#### 2.3.3 Dementia progression evaluation in ADNI

We evaluated the conversion to dementia probabilities of the newly obtained clusters from the ADNI cohort, including subjects with a diagnosis follow‐up until 10 years or more. For this, we performed survival analysis employing Cox proportional hazard (Cox‐PH) models and Kaplan–Meier curves using lifelines v0.27.0^19^ library from Python. We compared clustering results against the original AT(N) categories. Cox‐PH models included standard covariates such as subjects' sex, age at baseline, and education years. We considered significant *p*‐values less than 0.05.

### 2.4 Ethical statement

The study was conducted with the approval of our hospital Ethics Committee and participants (or their legally authorized representative) gave written informed consent.

## 3 RESULTS

### 3.1 Biomarkers clustering in HCSC and ADNI datasets

We obtained clusters for all CSF biomarkers combined in both HCSC and ADNI datasets. In the case of the HCSC dataset, we evaluated two datasets with different amyloid status representatives: one using Aβ(1–42) and another using Aβ(1–42)/Aβ(1–40) ratio. Tables S3 and S4 list the SI values obtained for each solution for HCSC and ADNI datasets, respectively.

The solutions that obtained the highest SI values in the HCSC dataset were *k =* 3 clusters and *k =* 2 clusters for combining all biomarkers using the Aβ(1–42)/Aβ(1–40) ratio and Aβ(1–42) as amyloid representatives, respectively. Given that the clustering solution using the Aβ(1–42)/Aβ(1–40) ratio gave significantly better SI scores than the Aβ(1–42) one, we decided to evaluate the first one in the HCSC dataset. In the case of ADNI, SI scores did not clearly show whether 2, 3, or 4 clusters were the best solution. Therefore, we decided to explore the three‐cluster solution of the ADNI dataset, evaluate the differences between cohorts, and evaluate the use of the ratio against the use of Aβ(1–42) jointly with other biomarkers. Therefore, we next describe the clustering results *k =* 3 for using Aβ(1–42)/Aβ(1–40) ratio in HCSC and Aβ(1–42) in ADNI cohorts.

Lastly, to properly generalize these three proposed groups, we evaluated the clustering results obtained in HCSC (discovery cohort) with another smaller cohort from ADNI (validation). The classification performance results were the following: 90.80% accuracy, 90.81% F1 score, and 84.43% MCC. Thus, meaning that the three‐cluster solution existed in both cohorts and these proposed CSF categories are generalizable.

### 3.2 Clusters description in HCSC and ADNI datasets

**FIGURE 1:**
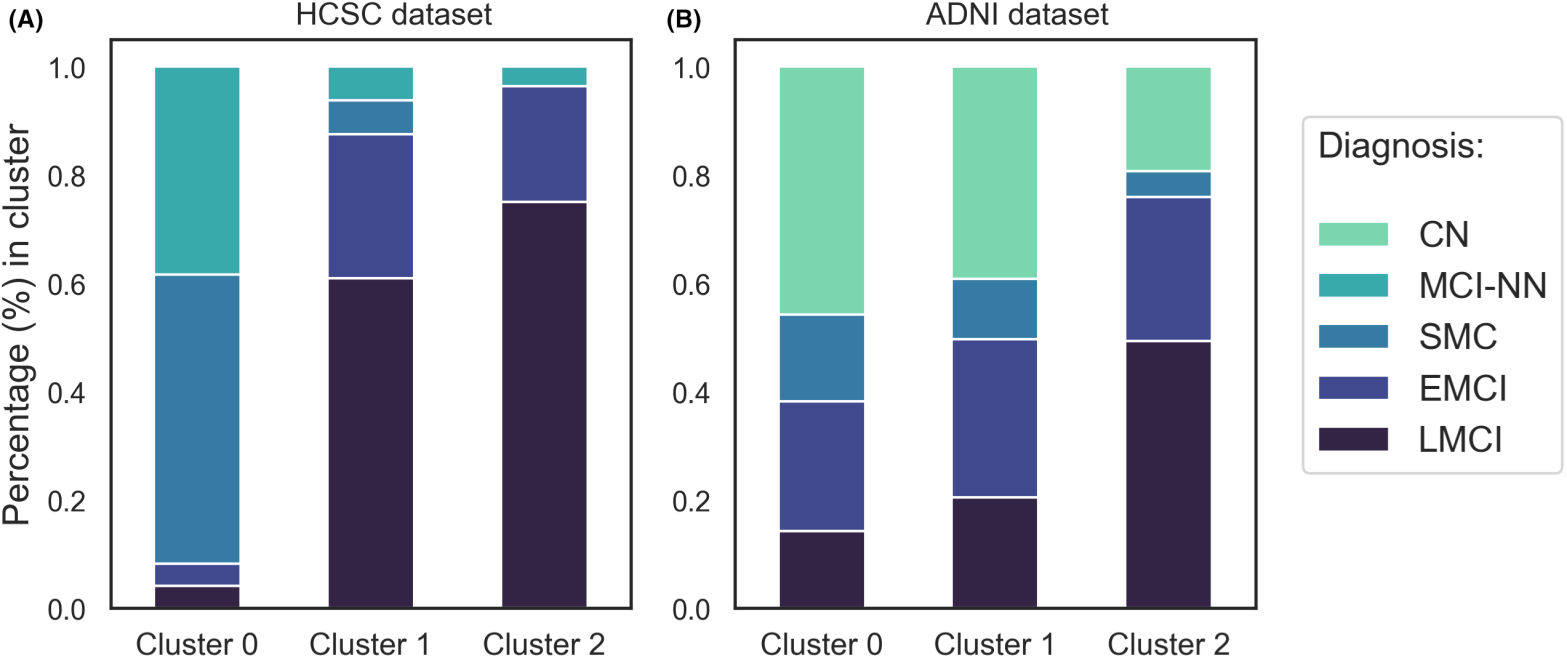
Stacked bar plots representing clinical diagnosis distribution (percentage, %) in each cluster. (A) HCSC dataset; (B) ADNI dataset. CN, Cognitively normal; MCI‐NN, mild cognitive impairment without neurodegeneration; SMC, subjective memory complaints; EMCI, early mild cognitive impairment; LMCI, late mild cognitive impairment.

Due to the unsupervised nature of clustering algorithms, it is necessary to analyze the solutions obtained from a clinical point of view beyond the score obtained from a computational perspective. We started describing the diagnosis signature of each obtained cluster. Figure 1 shows *within‐cluster* distribution of diagnoses in the HCSC and ADNI datasets.

The first cluster (Cluster 0) obtained in HCSC (Figure 1A) contained primarily SMC and MCI‐NN subjects. Clusters 1 and 2 presented the majority of MCI patients. Cluster 1 represents an intermediate group, with many more MCI cases than Cluster 0 but fewer than Cluster 2. Regarding the ADNI dataset (Figure 1B), we obtained similar results, although closer between clusters. In ADNI, the obtained intermediate cluster (Cluster 1), compared to HCSC's Cluster 1, was more similar to Cluster 0 in the CN and SMC subjects’ distribution. Thus, this intermediate cluster is more advanced in diagnosis in HCSC, while in ADNI, it is less advanced, more similar to Cluster 0. In addition, we noted that the distribution of EMCI cases remained nearly constant in all clusters (around 20%). On the contrary, LMCI, SMC, and CN changed greater their distributions among the different clusters. Finally, we observed that using Aβ(1–42)/Aβ(1–40) ratio rather than Aβ(1–42) as amyloid representative in combination with other CSF biomarkers yielded a much more sensitive output for Alzheimer's diagnosis (EMCI and LMCI) and specific for non‐Alzheimer's cases (SMC and MCI‐NN).

**FIGURE 2:**
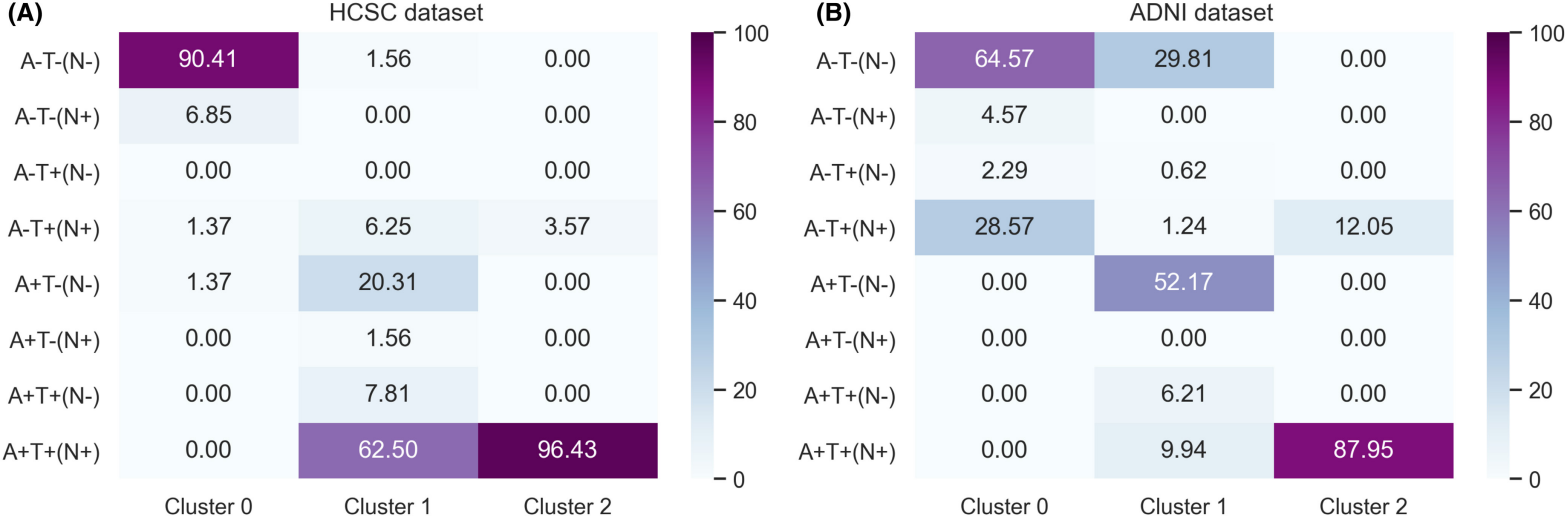
Heatmaps showing within‐cluster distribution (%) of different AT(N) categories in (A) HCSC and (B) ADNI datasets in clusters obtained with all biomarkers. Note that clustering results used different amyloid representatives: HCSC dataset used Aβ(1–42)/Aβ(1–40) ratio, whereas ADNI results used Aβ(1–42) value.

Next, we obtained the *within‐cluster* distribution of the original AT(N) categories (see Section 2.2 for the cut‐off values used for each dataset). Figure 2 shows heatmaps of these distributions for all biomarkers clustering results. In addition, Figure 2 shows AT(N) categories in a specific order, according to the AT(N) classification of the NIA‐AA^20^: A−T−(N−) (normal AD biomarkers); A−T−(N+) and A−T+(N±) (pathological change not AD type); A+T‐(N−) (AD continuum: Alzheimer‐like pathological change); A+T+(N±) (AD continuum: AD); and A+T(N+) (AD continuum: Alzheimer‐like pathological change with suspected pathology concomitant).

Regarding the HCSC dataset (Figure 2A), Cluster 0 mainly contained A−T−(N−) subjects (90.41%). Cluster 1 consisted almost entirely (92.18%) of A+ subjects, most of which were positive for all biomarkers (A+T+(N)+, 62.50%). This AT(N) category was followed by 20.31% of A+T−(N−) subjects. Finally, Cluster 2 consisted almost completely of A+T+(N)+ patients (96.43%). Regarding the ADNI dataset (Figure 2B), Cluster 0 was similar to Cluster 0 in HCSC. In the case of ADNI, Cluster 0 was formed entirely by A− subjects. However, compared to HCSC, there were more A− subjects with positive TN (28.57%). Cluster 1 mainly comprised A+ subjects (68.32%), a significantly lower percentage of A+ than Cluster 1 in the HCSC dataset (92.18%). Cluster 1 also contained around 30% of A− subjects. In Cluster 2, most subjects were positive for all biomarkers (87.95%), and a small percentage (12.05%) corresponded to A−T+(N)+ subjects. Finally, it is noteworthy that in HCSC and ADNI, we did not find either A−T−(N)+ or A+T−(N)+ subjects, respectively.

Continuing with the description of the clusters obtained by combining all biomarkers, we evaluated the main characteristics of each cluster, including CSF biomarkers, sex, age at baseline, education years, and MMSE score at baseline (depicted in Table 2).

**TABLE 2:** Clinical characteristics of clusters obtained in the HCSC and ADNI datasets.

Dataset	Variable	Cluster 0	Cluster 1	Cluster 2	Statistic	*p*‐Value
HCSC	Number	73	64	28	NA	NA
	Age (years) at baseline	66.44 ± 9.07	74.58 ± 7.29	73.57 ± 5.59	20.16	<0.001 *(a, b)*
	Sex, Female (%)	47.95	54.69	67.86	0.08	0.9591
	Education years	12.09 ± 4.69	9.69 ± 4.78	10.88 ± 5.08	3.85	0.0234
	MMSE at baseline	27.43 ± 2.72	25.26 ± 2.72	24.16 ± 4.22	10.04	<0.001 *(none)*
	Aβ(1–42)/Aβ(1–40) ratio	0.10 ± 0.01	0.05 ± 0.01	0.04 ± 0.02	441.33	<0.001 *(a, b, c)*
	pTau	34.64 ± 11.05	79.01 ± 24.62	173.31 ± 36.79	378.79	<0.001 *(a, b, c)*
	tTau	273.63 ± 93.41	516.19 ± 153.98	1060.36 ± 242.24	274.27	<0.001 *(a, b, c)*
ADNI	Number	175	161	83	*NA*	*NA*
	Age (years) at baseline	71.93 ± 6.60	73.17 ± 5.88	72.99 ± 7.71	1.65	0.1938
	Sex, Female (%)	49.14	42.86	46.99	0.01	0.9959
	Education years	16.15 ± 2.76	16.43 ± 2.61	15.88 ± 2.87	1.19	0.3046
	MMSE at baseline	28.78 ± 1.29	28.59 ± 1.47	27.55 ± 1.91	19.7	<0.001 *(b, c)*
	Aβ(1–42)	1612.92 ± 150.22	822.26 ± 277.78	769.26 ± 290.99	623.23	<0.001 *(a, b)*
	pTau	21.73 ± 5.68	17.71 ± 5.93	43.31 ± 11.77	350.95	<0.001 *(a, b, c)*
	tTau	248.46 ± 62.90	190.16 ± 56.10	425.09 ± 105.31	303.90	<0.001 *(a, b, c)*

*Note*: For continuous variables, values represent mean ± standard deviation. *p*‐values computed for ANOVA for continuous variables and the chi‐square test for continuous ones. ANOVA with Tukey post‐hoc analysis showed statistically significant differences between Clusters 0 and 1 (*a*), Clusters 0 and 2 (*b*), and Clusters 1 and 2 (*c*).

**FIGURE 3:**
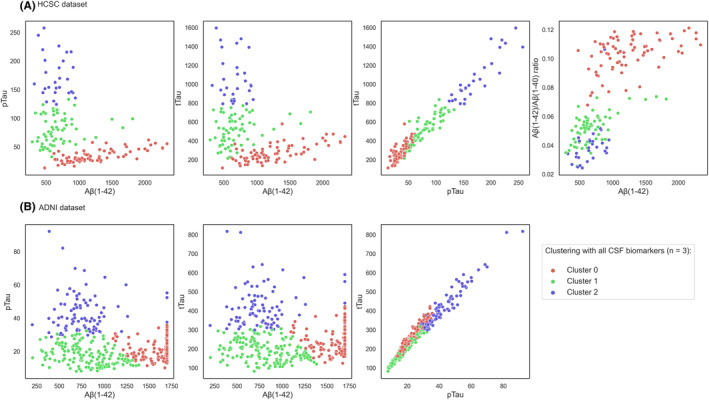
Scatterplots of CSF biomarkers values. Different colors represent cluster assignments in (A) HCSC and (B) ADNI datasets.

Concerning the mean values of the biomarkers, Figure 3 shows scatterplots of the CSF biomarker values to compare their distribution in the two datasets evaluated, according to the three clusters obtained with all biomarkers.

In both datasets, amyloid biomarkers were the most clearly defined differentiation between Cluster 0 versus Clusters 1 and 2. The mean values of the respective amyloid biomarkers shown in Table 2 also reflected this result, such as Cluster 0 > Cluster 1 > Cluster 2. In addition, regarding both datasets, the differentiation between Clusters 1 and 2 was defined by pTau or tTau. Figure 3 shows that the correlation between pTau and tTau in both datasets was very high, reflected in their respective representation against Aβ(1–42), which was practically the same.

However, the clustering results between datasets showed differences in the biomarkers' values distribution. Firstly, the values distribution of Aβ(1–42) against both pTau and tTau differed between datasets. In the case of HCSC, this distribution resembled more in an “L” shape (Figure 3A), whereas ADNI dataset biomarkers values were more dispersed (Figure 3B). Secondly, related to the previous result, the pTau and tTau means between Clusters 1 and 2 differed between datasets. On the one hand, in HCSC, while pTau and tTau values increased, they also increased their dispersion (or standard deviation). Table 2 also reflects this result, where the mean values and standard deviations of pTau and tTau meet the following progression: Cluster 2 > Cluster 1 > Cluster 0. On the other hand, regarding ADNI, pTau against tTau scatterplot (Figure 3B) and Table 2 showed pTau and tTau values in Cluster 0 were greater than Cluster 1, following distribution: Cluster 2 > Cluster 0 > Cluster 1.

### 3.3 Survival analysis of each biomarker clustering in ADNI

**FIGURE 4:**
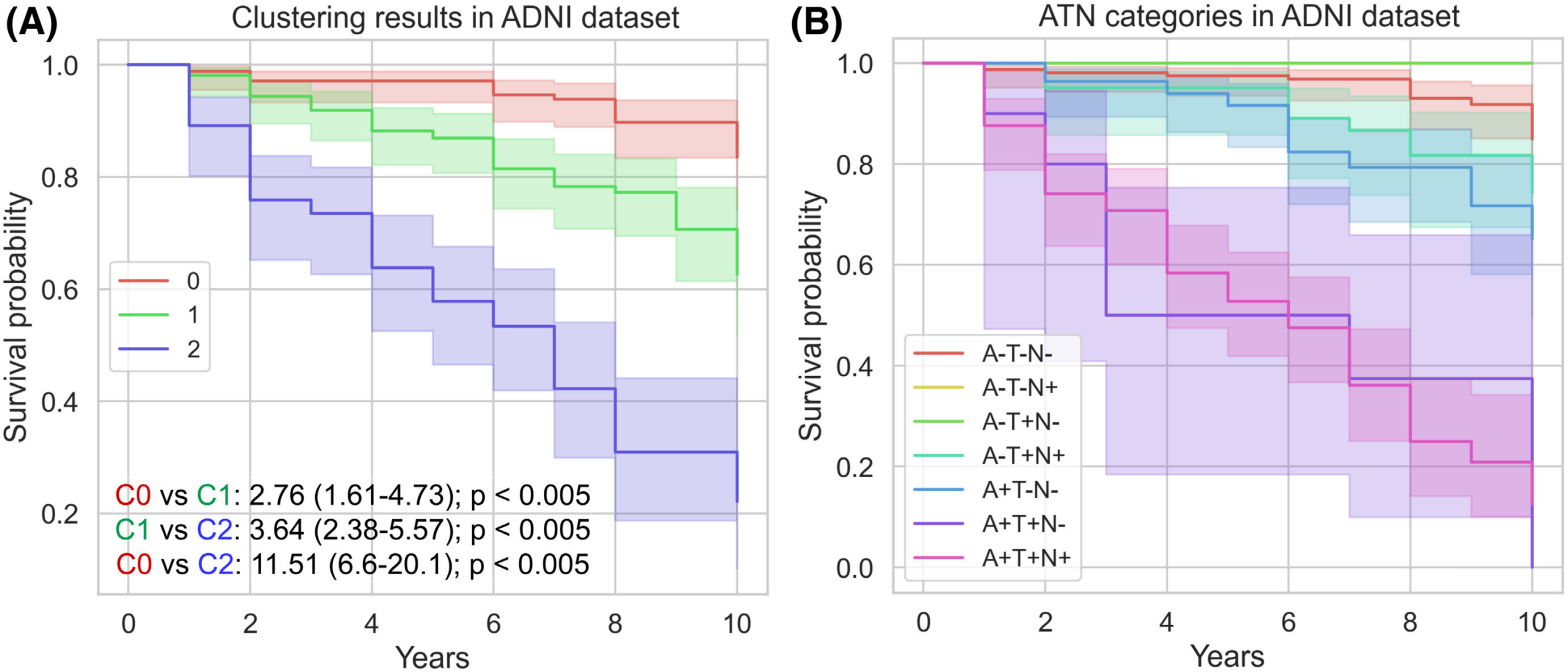
Kaplan–Meier curves and Cox‐PH models' results obtained in ADNI for (A) clustering results and (B) AT(N) categories. Cox‐PH models results are shown as hazard ratio (upper 95% CI–lower 95% CI); *p*‐value. Models were adjusted using covariates: age at baseline, years of education, and sex. C0: Cluster 0; C1: Cluster 1; C2: Cluster 2. For significant differences between AT(N) categories, please refer to Table S5.

Finally, we conducted survival analyses in ADNI to assess whether the new clusters showed significant differences in dementia progression compared to the AT(N) categories. Figure 4 shows the Kaplan–Meier curves of the clusters and the hazard ratios (HR) and *p*‐values obtained.

The three clusters in ADNI showed significant (*p*‐values <0.005) differences regarding progression to dementia, even between Clusters 1 and 2 (Figure 4A). Cluster 2 represented the most affected, with an increased risk of dementia of 3.64 and 11.51 compared to Clusters 0 and 1, respectively. Regarding the categories of the AT(N) system (Figure 4B), Table S5 shows the HRs and *p*‐values obtained when comparing all AT(N) categories present by pairs (in the absence of A+T−[N+] individuals). Seven of twenty‐one (33%) AT(N) comparisons resulted in statistical significance (*p*‐value <0.005). Among these pairs, five of seven (71.43%) showed increased risk when A biomarker was positive (A−T+[N+] vs. A+T+[N‐] and A+T+N+; A−T−[N−] vs. A+T+[N−], A+T−[N−], and A+T+[N+]). The remaining AT(N) pairs showed an increased risk when T or TN biomarkers were positive in the presence of A+ (A+T+[N−] vs. A+T−[N−]; A+T−[N−] vs. A+T+[N+]). The remaining AT(N) pairs overlapped and were not significantly different for the progression to dementia.

## 4 DISCUSSION

In this study, we conducted a data‐driven strategy based on clustering to search for new groups in the combination of common CSF biomarkers (Aβ(1–42)/Aβ(1–40) ratio, Aβ(1–42), pTau, and tTau). We evaluated how the information of all the biomarkers combined could result in different clustering solutions in two cohorts. Lastly, we compared the optimal clustering solutions against the currently employed AT(N) system categories to assess differences between both methods.

Importantly, two main methodological differences between the two cohorts employed need to be discussed. First, the amyloid representative: Aβ(1–42)/Aβ(1–40) and Aβ(1–42) in the HCSC and ADNI cohorts, respectively. The ratio differentiated patients better with AD and, as in the AT(N) system, appeared to be a more appropriate biomarker to carry out unsupervised strategies for patient stratification. Second, how patients were recruited in each cohort. The HCSC cohort was obtained from clinical practice, in which the majority of subjects included were patients who came with at least subjective memory complaints or cognitive impairment and were therefore expected to be slightly more advanced in the disease than the ADNI cohort. On the contrary, the ADNI cohort is a research cohort in which many more CN subjects appear at the initial visit since it aims to represent the potential deterioration of these patients in a longitudinal study. Despite these methodological differences, we believe that the combination of both cohorts and the replication of the findings in both settings was important to confirm the external validity of the presented study.

One of the key points in this work, compared with the AT(N) system, is that cut‐off points are established separately for each biomarker in the latter. Then, joint categories are created without taking into account the interactions that may exist between biomarkers. This dichotomization of biomarkers in the AT(N) system shows inconsistencies when combining different biomarkers^8^ and loses prognostic ability compared to using them on a continuous basis.^21^ Moreover, clustering techniques like the one applied in this work would not require determining the cut‐off points using traditional methods. This fact avoids the need for a comparison with a healthy control group (which could be in preclinical stages and develop AD in the following years) or using amyloid PET (which also requires cut‐off points) as standard.^17^, ^22^, ^23^, ^24^ Moreover, it considers the mentioned interaction between biomarkers. Thus, clustering techniques could be useful to obtain valid groups for different cohorts, reducing the variability across centers and the limitations of using cut‐off points from other populations^25^, ^26^, ^27^ and with a similar computational cost to the AT(N) system.

Interestingly, our findings suggest clustering CSF biomarkers together, especially when using Aβ(1–42)/Aβ(1–40) ratio as the amyloid representative, led to three clusters. These three clusters appeared in the two cohorts and presented differences in the distribution of the clinical diagnoses (Figure 1), AT(N) categories (Figure 2), biomarker values distribution (Table 2 and Figure 3), and survival (Figure 4). Accordingly, we propose three categories for the AD diagnosis based on CSF biomarker values combined: (i) not affected by AD, (ii) AD in a very early stage or slow progression, and (iii) AD in a more advanced stage or more rapid progression. Our results are consistent with another clustering work^28^ in an independent cohort of 151 patients with AD, in which a group with higher levels of Tau and lower amyloid levels was associated with worse clinical outcomes over time, including faster cognitive decline and higher mortality.

The first cluster included individuals with high beta‐amyloid and low pTau and tTau levels, consisting mostly of individuals with no AD (A−T−[N−]) or, especially in the case of ADNI, subjects with a pathological change not associated with AD (A−T+[N±] or A−T−[N+]). The second cluster consisted of an intermediate or transitional group, with most individuals having a low beta‐amyloid biomarker value and high Tau levels. This intermediate group was the one that showed the greatest differences between the two cohorts. In HCSC, the intermediate cluster was more similar to the most affected (Cluster 2), with amyloid positivity but lower tau levels. ADNI's intermediate cluster was also positive for amyloid but showed Tau levels closer to the least affected cluster (Cluster 0). Finally, the third cluster included subjects with very low beta‐amyloid values and extremely high pTau and tTau. Nearly all A+T+(N+) subjects were in the third cluster in both datasets.

The results showed amyloid categorization is dichotomous, as proposed by the AT(N) system.^6^ Furthermore, as other works have previously suggested,^29^ a much clearer and cleaner binary categorization of patients happens when using Aβ(1–42)/Aβ(1–40) ratio rather than Aβ(1–42) alone. Therefore, it is advisable to use the former whenever possible. Moreover, we found that Tau biomarkers are not dichotomous but rather continuous, significantly different among the three clusters obtained in the two cohorts (Table 2). This fact was also reflected in the SI scores obtained for these biomarkers alone (Tables S3 and S4), which were very similar, probably making tTau a “redundant” biomarker when using CSF biomarkers in the context of AD.

A counterintuitive result is that Tau is a stronger predictor for HCSC than ADNI (Figure 3). One potential first reason could be the different amyloid representatives employed in each case. The amyloid ratio value in the HCSC cohort seemed to provide more information for the characterization of Cluster 0 versus Clusters 1 and 2. Then, HCSC's Clusters 1 and 2 could be better characterized using tau values, acquiring more importance in the proposed classification. This fact most likely caused the clusters not to be defined in the same way in both scenarios, generating the phenomenon that can be observed in Figure 3. Second, and importantly, we found that Aβ(1–42) presented many saturated points at value 1700 in the ADNI cohort. Another work performed on ADNI included these saturated points as extrapolated values,^17^ resulting in a much more similar distribution as the one presented for HCSC in Figure 3. We did not have access to these extrapolated values and straightly replaced them with the value of 1700, thus, clearly affecting clustering results. When we performed the clustering eliminating the patients presenting this extreme value, we obtained much more similar results to those obtained in the case of HCSC, with pTau and tTau getting higher values according to the cluster progression (Table S6).

Importantly, we performed a clustering‐then‐classification analysis to corroborate the three clusters found in both cohorts. This classification model developed is the one we present for classifying new patients in the proposed CSF categories. In the future, it will be necessary to train it with a larger number of samples, evaluating and comparing the results of more and different cohorts. Moreover, the great classification performance obtained (more than 90% accuracy) corroborated the presence of three clusters in both the HCSC cohort (discovery dataset) and in another ADNI cohort that included the amyloid ratio value (validation dataset). Furthermore, and notably, the original ADNI cohort, which did not contain the amyloid ratio, also showed grouping in three similar clusters, which were significantly different regarding their survival.

The survival analysis performed on clustering results from ADNI (Figure 4) validates the clustering as a data‐driven tool for the prognosis of AD. The three obtained clusters showed a significant difference in the progression of dementia. Conversely, when comparing survival between different AT(N) categories, we found that only 33% of AT(N) categories significantly differed in the progression to dementia. The remaining AT(N) categories were mixed in survival, similar to recent studies.^30^ Overall, our findings suggest that, when employing CSF biomarkers, it could not be necessary to use a fine‐grained categorization such as the AT(N) system proposes. In addition, the AT(N) categories, because they are assigned independently and separately, may lose information on the relationships between the biomarkers, which is relevant as Tau and amyloid represent key linked pathophysiological processes.

Our study presents some limitations. First, survival analysis was only evaluated on the ADNI cohort because data from HCSC had a shorter follow‐up time. Second, we only evaluated a single clustering algorithm, KMeans, as it is a standard and widely used method, especially using low‐dimensionality (few input variables) datasets. In the future, it would be interesting to corroborate the results obtained with clustering methods that also consider non‐linear relationships on the different biomarkers and explore whether more clinically relevant clusters appear. Another clustering work using CSF biomarkers applied a Gaussian mixture modeling algorithm, which yielded six clusters.^31^ However, this work included more non‐AD pathologies and did not report survival differences between the clusters. Third, we only focused on certain CSF biomarkers to increase the applicability of our findings in clinical settings, where biomarkers from multiple sources are generally absent. Other works suggest that including more biomarkers, such as MRI measures^32^ or additional CSF biomarkers,^33^ will lead to more clusters. Recent studies have proposed to split the “T” category of the AT(N) system into CSF pTau and tau‐PET.^34^ In this regard, combining CSF with other blood biomarkers and MRI and PET neuroimaging would be of interest in future studies using unsupervised machine learning approaches. Another limitation regarding including new inputs in the model is that we did not include sociodemographic variables such as age or education years in the proposed clustering models. Clustering results showed that lower amyloid values were related to younger age. Therefore, in the future, it would be necessary to develop these classification models, including sociodemographic features, and assess their effects on the output of clusters similar to those we proposed. Finally, although we obtained a good classification performance, the subjects that were more difficult to classify were those in an intermediate cluster with mixed biomarker characteristics, for example, amyloid‐positive and tau‐negative. This result suggests that the classification model we proposed still requires further improvement. In the future, the inclusion of additional cohorts with longitudinal information to study each cluster's clinical evolution, especially the mentioned mixed groups, is necessary.

Overall, our results showed that KMeans clustering analysis of biomarkers together obtains three categories according to common CSF biomarkers: (i) subjects with high Aβ(1–42) and low pTau and tTau values non‐defined or unrelated to AD, (ii) subjects with low Aβ(1–42) and high pTau and tTau values, representing early stages and/or a more delayed risk of conversion to dementia, and (iii) subjects with low Aβ(1–42) and extremely high pTau and tTau values, associated with more severe cognitive impairment within the early stages of AD and with more rapid progression to dementia. In addition, we confirm the better properties of the Aβ(1–42)/Aβ(1–40) ratio compared with Aβ(1–42) as a biomarker of amyloidosis in CSF. This three‐group classification, developed with a data‐driven approach, may be obtained without the need to use cut‐off points and represents a more meaningful and straightforward approach to evaluate the risk of conversion to dementia that may be complementary to the AT(N) system classification. These findings suggest that the joint interpretation of several biomarkers based on machine learning techniques may improve the outcome prediction in the AD continuum.

## AUTHOR CONTRIBUTIONS

LH‐L, MJG‐M, JAM‐G, and JLA: conceptualization and design of the work. MJG‐M, JM‐G, and JAM‐G: methodology. LH‐L, MJG‐M, JAM‐G, IO, MCC, MD‐C, AD‐A, and MP‐S: data curation. LH‐L: formal analysis and software development. All: investigation and supervision. JM‐G, JLA, and JAM‐G: funding acquisition. LH‐L: visualization. MJG‐M and JAM‐G: project administration. MJG‐M and JAM‐G: resources. LH‐L, MJG‐M, and JAM‐G: original draft writing. JM‐G, IO, MCC, MD‐C, AD‐A, MP‐S, and SC: writing review and editing. All authors contributed to the article and approved the submitted version.

## FUNDING INFORMATION

LH‐L. is supported by a predoctoral grant from Complutense University of Madrid and Banco Santander [grant number CT63/19‐CT64/19]. J.L.A. is supported by the Spanish Ministry of Science and Innovation under project PID2019‐110866RB‐I00. J.A.M.‐G. is supported by Instituto de Salud Carlos III through project INT20/00079 (co‐funded by the European Regional Development Fund “A way to make Europe”). MDC is supported by a Sara Borrell postdoctoral fellowship from the Instituto de Salud Carlos III (CD22/00043) (co‐funded by European Regional Development Fund “A way to make Europe”).

## CONFLICT OF INTEREST STATEMENT

The authors declare that they have no competing interests.

## Supporting information


Appendix S1

## Data Availability

All the analyses and methods described here were implemented in Python v.3.8.12. The code developed for this work is available at https://github.com/laurahdezlorenzo/CSF_clustering. The ADNI dataset supporting the conclusions of this article is available under formal request at adni.loni.usc.edu. The HCSC data used and analyzed during the current study are available from the corresponding author upon reasonable request.

## References

[1] Schneider L . Alzheimer's disease and other dementias: update on research. Lancet Neurol. 2017;16(1):4‐5. doi:10.1016/S1474-4422(16)30356-8 27979354

[2] Hadjichrysanthou C , Evans S , Bajaj S , et al. The dynamics of biomarkers across the clinical spectrum of Alzheimer's disease. Alzheimers Res Ther. 2020;12(1):74. doi:10.1186/s13195-020-00636-z 32534594 PMC7293779

[3] Albert MS , DeKosky ST , Dickson D , et al. The diagnosis of mild cognitive impairment due to Alzheimer's disease: recommendations from the National Institute on Aging‐Alzheimer's Association workgroups on diagnostic guidelines for Alzheimer's disease. Alzheimers Dement J Alzheimers Assoc. 2011;7(3):270‐279. doi:10.1016/j.jalz.2011.03.008 PMC3312027 21514249

[4] Petersen RC , Smith GE , Waring SC , Ivnik RJ , Tangalos EG , Kokmen E . Mild cognitive impairment: clinical characterization and outcome. Arch Neurol. 1999;56(3):303‐308. doi:10.1001/archneur.56.3.303 10190820

[5] d'Abramo C , D'Adamio L , Giliberto L . Significance of blood and cerebrospinal fluid biomarkers for Alzheimer's disease: sensitivity, specificity and potential for clinical use. J Pers Med. 2020;10(3):E116. doi:10.3390/jpm10030116 PMC7565390 32911755

[6] Jack CR , Bennett DA , Blennow K , et al. A/T/N: an unbiased descriptive classification scheme for Alzheimer disease biomarkers. Neurology. 2016;87(5):539‐547. doi:10.1212/WNL.0000000000002923 27371494 PMC4970664

[7] Zeng HM , Han HB , Zhang QF , Bai H . Application of modern neuroimaging technology in the diagnosis and study of Alzheimer's disease. Neural Regen Res. 2021;16(1):73‐79. doi:10.4103/1673-5374.286957 32788450 PMC7818875

[8] Illán‐Gala I , Pegueroles J , Montal V , et al. Challenges associated with biomarker‐based classification systems for Alzheimer's disease. Alzheimers Dement Diagn Assess Dis Monit. 2018;10:346‐357. doi:10.1016/J.DADM.2018.03.004 PMC6114028 30175226

[9] Altomare D , Wilde A d , Ossenkoppele R , et al. Applying the ATN scheme in a memory clinic population: the ABIDE project. Neurology. 2019;93(17):e1635‐e1646. doi:10.1212/WNL.0000000000008361 31597710

[10] Beam AL , Kohane IS . Big data and machine learning in health care. JAMA. 2018;319(13):1317‐1318. doi:10.1001/jama.2017.18391 29532063

[11] Kononenko I . Machine learning for medical diagnosis: history, state of the art and perspective. Artif Intell Med. 2001;23(1):89‐109. doi:10.1016/S0933-3657(01)00077-X 11470218

[12] Van Calster B , Wynants L . Machine learning in medicine. N Engl J Med. 2019;380(26):2588‐2590. doi:10.1056/NEJMc1906060 31242379

[13] Habes M , Grothe MJ , Tunc B , McMillan C , Wolk DA , Davatzikos C . Disentangling heterogeneity in Alzheimer's disease and related dementias using data‐driven methods. Biol Psychiatry. 2020;88(1):70‐82. doi:10.1016/j.biopsych.2020.01.016 32201044 PMC7305953

[14] Herukka SK , Simonsen AH , Andreasen N , et al. Recommendations for cerebrospinal fluid Alzheimer's disease biomarkers in the diagnostic evaluation of mild cognitive impairment. Alzheimers Dement J Alzheimers Assoc. 2017;13(3):285‐295. doi:10.1016/j.jalz.2016.09.009 28341066

[15] Wallin A , Nordlund A , Jonsson M , et al. The Gothenburg MCI study: design and distribution of Alzheimer's disease and subcortical vascular disease diagnoses from baseline to 6‐year follow‐up. J Cereb Blood Flow Metab. 2016;36(1):114‐131. doi:10.1038/jcbfm.2015.147 26174331 PMC4758548

[16] Blennow K , Shaw LM , Stomrud E , et al. Predicting clinical decline and conversion to Alzheimer's disease or dementia using novel Elecsys Aβ(1–42), pTau and tTau CSF immunoassays. Sci Rep. 2019;9(1):19024. doi:10.1038/s41598-019-54204-z 31836810 PMC6911086

[17] Hansson O , Seibyl J , Stomrud E , et al. CSF biomarkers of Alzheimer's disease concord with amyloid‐β PET and predict clinical progression: a study of fully automated immunoassays in BioFINDER and ADNI cohorts. Alzheimers Dement. 2018;14(11):1470‐1481. doi:10.1016/j.jalz.2018.01.010 29499171 PMC6119541

[18] Pedregosa F , Varoquaux G , Gramfort A , et al. Scikit‐learn: machine learning in python. J Mach Learn Res. 2011;12:2825‐2830.

[19] Davidson‐Pilon C . Lifelines: survival analysis in python. J Open Source Softw. 2019;4(40):1317. doi:10.21105/joss.01317

[20] Jack CR , Bennett DA , Blennow K , et al. NIA‐AA research framework: toward a biological definition of Alzheimer's disease. Alzheimers Dement J Alzheimers Assoc. 2018;14(4):535‐562. doi:10.1016/j.jalz.2018.02.018 PMC5958625 29653606

[21] Mattsson‐Carlgren N , Leuzy A , Janelidze S , et al. The implications of different approaches to define AT(N) in Alzheimer disease. Neurology. 2020;94(21):E2233‐E2244. doi:10.1212/WNL.0000000000009485 32398359 PMC7357296

[22] Leitão MJ , Silva‐Spínola A , Santana I , et al. Clinical validation of the Lumipulse G cerebrospinal fluid assays for routine diagnosis of Alzheimer's disease. Alzheimers Res Ther. 2019;11(1):91. doi:10.1186/s13195-019-0550-8 31759396 PMC6875031

[23] Lombardi G , Pupi A , Bessi V , et al. Challenges in Alzheimer's disease diagnostic work‐up: amyloid biomarker incongruences. J Alzheimers Dis. 2020;77(1):203‐217. doi:10.3233/JAD-200119 32716357

[24] Dumurgier J , Sabia S , Zetterberg H , et al. A pragmatic, data‐driven method to determine cutoffs for CSF biomarkers of Alzheimer disease based on validation against PET imaging. Neurology. 2022;99(7):e669‐e678. doi:10.1212/WNL.0000000000200735 35970577 PMC9484605

[25] Bayart JL , Hanseeuw B , Ivanoiu A , van Pesch V . Analytical and clinical performances of the automated Lumipulse cerebrospinal fluid Aβ42 and T‐Tau assays for Alzheimer's disease diagnosis. J Neurol. 2019;266(9):2304‐2311. doi:10.1007/s00415-019-09418-6 31179518

[26] Delaby C , Muñoz L , Torres S , et al. Impact of CSF storage volume on the analysis of Alzheimer's disease biomarkers on an automated platform. Clin Chim Acta Int J Clin Chem. 2019;490:98‐101. doi:10.1016/j.cca.2018.12.021 30579960

[27] Moon S , Kim S , Mankhong S , et al. Alzheimer's cerebrospinal biomarkers from Lumipulse fully automated immunoassay: concordance with amyloid‐beta PET and manual immunoassay in Koreans: CSF AD biomarkers measured by Lumipulse in Koreans. Alzheimers Res Ther. 2021;13(1):22. doi:10.1186/s13195-020-00767-3 33436035 PMC7802266

[28] Wallin ÅK , Blennow K , Zetterberg H , Londos E , Minthon L , Hansson O . CSF biomarkers predict a more malignant outcome in Alzheimer disease. Neurology. 2010;74(19):1531‐1537. doi:10.1212/WNL.0B013E3181DD4DD8 20458070

[29] Hansson O , Lehmann S , Otto M , Zetterberg H , Lewczuk P . Advantages and disadvantages of the use of the CSF Amyloid β (Aβ) 42/40 ratio in the diagnosis of Alzheimer's disease. Alzheimers Res Ther. 2019;11(1):34. doi:10.1186/s13195-019-0485-0 31010420 PMC6477717

[30] Ebenau JL , Timmers T , Wesselman LMP , et al. ATN classification and clinical progression in subjective cognitive decline: the SCIENCe project. Neurology. 2020;95(1):e46. doi:10.1212/WNL.0000000000009724 32522798 PMC7371376

[31] Bellomo G , Indaco A , Chiasserini D , et al. Machine learning driven profiling of cerebrospinal fluid core biomarkers in Alzheimer's disease and other neurological disorders. Front Neurosci. 2021;15:337. doi:10.3389/FNINS.2021.647783/BIBTEX PMC8044304 33867925

[32] Racine AM , Koscik RL , Berman SE , et al. Biomarker clusters are differentially associated with longitudinal cognitive decline in late midlife. Brain. 2016;139(8):2261‐2274. doi:10.1093/BRAIN/AWW142 27324877 PMC4958904

[33] Toschi N , Lista S , Baldacci F , et al. Biomarker‐guided clustering of Alzheimer's disease clinical syndromes. Neurobiol Aging. 2019;83:42‐53. doi:10.1016/J.NEUROBIOLAGING.2019.08.032 31585366

[34] Groot C , Smith R , Stomrud E , et al. Phospho‐tau with subthreshold tau‐PET predicts increased tau accumulation rates in amyloid‐positive individuals. Brain. 2022;146(4):1580‐1591. doi:10.1093/brain/awac329/6695020 PMC10115173 36084009

