# Supplementary material for: A data‐driven approach to complement the A/T/(N) classification system using CSF biomarkers
Source: CNS Neurosci Ther. 2023 Jul 27;30(2):e14382. doi: 10.1111/cns.14382 (PMC10848077; doi:10.1111/cns.14382)
Supplement: Supplementary file 1 — Appendix S1 [file CNS-30-e14382-s001.docx]

### Supplementary Information

#### 1. Materials and Methods

**1.1. HCSC dataset neuropsychological protocol**

A comprehensive neuropsychological assessment was administered and used for diagnosis and classification. This protocol included the following test: Mini-Mental State Examination (MMSE) [1], Addenbrooke's Cognitive Examination III (ACE-III) [2], digit span forward and backward, Corsi’s test forward and backward, Trail Making Test (TMT) (parts A and B), Symbol Digit Modalities Test (SDMT), Stroop Color-Word Interference test (word reading, color naming, and interference, SCWIT), Tower of London-Drexel version (ToL), Boston Naming Test (BNT), Free and Cued Selective Reminding Test (FCSRT), Rey-Osterrieth Complex Figure (copy, memory at 3 and 30 minutes, and recognition) (ROCF), Judgment of Line Orientation (JLO), and the Visual Object and Space Perception Battery (VOSP) (subtests object decision, progressive silhouettes, discrimination of position, and number location). These tests belonged to the Neuronorma battery and were used to study the potential impairment of other cognitive domains beyond verbal memory using the normative data from the Neuronorma study [3-9].

Furthermore, we assessed functional independence using the basic and instrumental activities of daily living questionnaires: Pfeffer Functional Activities Questionnaire (FAQ) [10] and Interview for Deterioration in Daily life in Dementia (IDDD) [11].

**1.2. HCSC dataset CSF samples acquisition and analysis**

CSF samples were collected between December 2018 and December 2021 as a part of the subject´s routine clinical diagnosis investigation. Pre-analytical and analytical procedures were done in accordance with previously proposed protocols [12]. CSF samples were collected in 10 mL sterile polypropylene tubes (Sarstedt, Ref#62.610.201), centrifuged within 2 hours at 2000g for 10 minutes and room temperature, and aliquoted into 2 mL polypropylene tubes (Sarstedt, Ref#72.694.007), and stored at -80ºC until analysis.

Samples were analyzed for the four markers (β-Amyloid 1-42, β-Amyloid 1-40, total Tau, and pTau) directly from the storage tubes containing 1 mL of CSF using the Lumipulse G β-Amyloid 1-42, β-Amyloid 1-40, tTau and pTau181 assays on LUMIPULSE G600II automated platform (Fujirebio) and following the manufacturer's instructions. Buffer-based quality control testing was performed at the beginning of each test day to ensure that all measured values of each control level (low, medium, and high) were within the target ranges. The same batch of reagents for each marker/assay was used throughout the method comparison study.

The results of the Luminpulse β-Amyloid 1-42 presented in this study have been standardized according to a certified reference material developed by the International Federation of Clinical Chemistry and Laboratory Medicine as recommended by their working group for CSF proteins [13]. Briefly, values of the calibration standards of the Lumipulse G β-Amyloid 1-42 were adapted to the certified reference material (CRM), resulting in an adjustment of concentrations that were linearly proportional throughout the range. The aim of standardization to CRM is to harmonize immunoassays of Aβ42 to make results comparable across different platforms.

References:

1. M.F. Folstein, S.E. Folstein, P.R. McHugh.«Mini-mental state». A practical method for grading the cognitive state of patients for the clinician. J Psychiatr Res., 12 (1975), pp. 189-198.
2. Matías-Guiu JA, Fernández-Bobadilla R, Fernández-Oliveira A, Valles-Salgado M, Rognoni T, Cortés-Martínez A, Moreno-Ramos T, Kulisevsky J, Matías-Guiu J. Normative Data for the Spanish Version of the Addenbrooke's Cognitive Examination III. .Dement Geriatr Cogn Disord. 2016;41(5-6):243-50.
3. Peña-Casanova J, Blesa R, Aguilar M, Gramunt-Fombuena N, Gómez-Ansón B, Oliva R, Molinuevo JL, Robles A, Bar-quero MS, Martínez-Parra C, Frank-García A, Fernández M, Antúnez C, Alfonso V, Sol JM; NEURONORMA Study team (2009) Spanish Multicenter Normative Studies (NEU RONORMA Project): Methods and sample characteristics. Arch Clin Neuropsychol 24, 307–319. [PubMed: 19549723]
4. Peña-Casanova J, Gramunt-Fombuena N, Quiñones-úbeda S, Sánchez-Benavides G, Aguilar M, Badenes D, Molinuevo JL, Robles A, Barquero MS, Payno M, Antunez C, Martínez-Parra C, Frank-García A, Fernández M, Alfonso V, Sol JM, Blesa R, for the NEURONORMA, Study Team (2009) Spanish Multicenter Normative Studies (NEURONORMA Project): Norms for the Rey-Osterrieth Complex Figure (Copy and Memory), and Free and Cued Selective Reminding Test. Arch Clin Neuropsychol 24, 371–393. [PubMed: 19661107]
5. Peña-Casanova J, Quiñones-úbeda S, Gramunt-Fombuena N, Aguilar M, Casas L, Molinuevo JL, Robles A, Rodríguez D, Martínez-Parra C, Frank-García A, Barquero MS, Antunez A, Fernández M, Molano A, Alfonso V, Sol JM, Blesa R, Study NEURONORMA, Team (2009) Spanish Multicenter Normative Studies (NEURONORMA Project): Norms for Boston naming test and token test. Arch Clin Neuropsychol 24, 343–354. [PubMed: 19648582]
6. Peña-Casanova J, Quiñones-úbeda S, Gramunt-Fombuena N, Quintana-Aparicio M, Aguilar M, Badenes D, Cerulla N, Molinuevo JL, Ruiz E, Robles A, Barquero MS, Antúnez C, Martínez-Parra C, Frank-García A, Fernández M, Alfonso V, Sol JM, Blesa R, Study NEURONORMA, Team (2009) Spanish Multicenter Normative Studies (NEURONORMA Project): Norms for verbal fluency tests. Arch Clin Neuropsychol 24, 395–411.
7. Peña-Casanova J, Quintana-Aparicio M, Quiñones-úbeda S, Aguilar M, Molinuevo JL, Serradell M, Robles A, Bar-quero MS, Martínez-Parra C, Villanueva C, Antunez Frank-García A, Aguilar MD, Fernández M, Alfonso V, Sol JM, Blesa R, Study NEURONORMA, Team (2009) Spanish Multicenter Normative Studies (NEURONORMA Project): Norms for the visual object and space perception battery-abbreviated, and judgement of line orientation. Arch Clin Neuropsychol 24, 355–370. [PubMed: 19648584]
8. Peña-Casanova J, Quiñones-úbeda S, Gramunt-Fombuena N, Quintana M, Aguilar M, Molinuevo JL, Serradell M, C, Martínez-Robles A, Barquero MS, Payno M, Antunez Parra C, Frank-García A, Fernández M, Alfonso V, Sol JM, Blesa R, Study NEURONORMA, Team (2009) Spanish Multicenter Normative Studies (NEURONORMA Project): Norms for the Stroop color-word interference test and the Tower of London-Drexel. Arch Clin Neuropsychol 24, 413–429. [PubMed: 19661108]
9. Peña-Casanova J, Quiñones-úbeda S, Quintana-Aparicio M, Aguilar M, Badenes D, Molinuevo JL, Torner L, Robles C, Martínez-Parra A, Barquero MS, Villanueva C, Antunez C, Frank-García A, Sanz A, Fernández M, Alfonso V, Sol JM, Blesa R, Study NEURONORMA, Team (2009) Spanish Multicenter Normative Studies (NEURONORMA Project): Norms for verbal span, visuospatial span, letter and number sequencing, trail making test, and symbol digit modalities test. Arch Clin Neuropsychol 24, 321–341. [PubMed: 19661109]
10. R.I. Pfeffer, T.T. Kurosaki, C.H. Harrah Jr., J.M. Chance, S. Filos. Measurement of functional activities in older adults in the community. J Gerontol, 37 (1982), pp. 323-329
11. Teunisse S, Derix MM. Meten van het dagelijks functionerenvan thuiswonende dementie patienten: ontwikkeling van eenvragenlijst. Tijdschr Gerontol Geriatr 1991; 22: 53-59.
12. Del Campo M, Mollenhauer B, Bertolotto A, Engelborghs S, Hampel H, Simonsen AH, Kapaki E, Kruse N, Le Bastard N, Lehmann S, Molinuevo JL, Parnetti L, Perret-Liaudet A, Sáez-Valero J, Saka E, Urbani A, Vanmechelen E, Verbeek M, Visser PJ, Teunissen C. Recommendations to standardize preanalytical confounding factors in Alzheimer’s and Parkinson’s disease cerebrospinal fluid biomarkers: an update. Biomark Med. 2012;6:419–30. <https://doi.org/10.2217/bmm.12.46>.
13. Kuhlmann J, Andreasson U, Pannee J, Bjerke M, Portelius E, Leinenbach A, Bittner T, Korecka M, Jenkins RG, Vanderstichele H, Stoops E, Lewczuk P, Shaw LM, Zegers I, Schimmel H, Zetterberg H, Blennow K; IFCC Working Group on Standardization of CSF proteins (WG-CSF). CSF Aβ 1–42 – an excellent but complicated Alzheimer’s biomarker – a route to standardisation. Clin Chim Acta 2017;467:27–33. doi:<https://doi.org/10.1016/j.cca.2016.05.014>

**Supplementary Table 1.** HCSC dataset diagnostic criteria.

|  | LMCI | EMCI | SMC | MCI-NN |
| --- | --- | --- | --- | --- |
| First visit | Subjective memory complaints | Subjective memory complaints | Subjective memory complaints | Subjective memory complaints |
| Cognitive status evaluation | FCSRT (delayed recall or total recall scaled score) **≤ 6**  No significant impairment in social and occupational function that would merit a diagnosis of major neurocognitive  disorder or dementia | FCSRT (delayed recall or total recall scaled score) **≥ 7**  No significant impairment in social and occupational function that would merit a diagnosis of major neurocognitive  disorder or dementia | FCSRT (delayed recall or total recall scaled score) **≥ 7**  No significant impairment in social and occupational function that would merit a diagnosis of major neurocognitive  disorder or dementia | FCSRT (delayed recall or total recall scaled score) **≤ 6** |
| AD biomarker support (*) | At least one | At least one | None | None |

LMCI: Late Mild Cognitive Impairment, EMCI: Early Mild Cognitive Impairment, SMC: Subjective Memory Complaint; MCI-NN: MCI without evidence of neurodegeneration

FCSRT: Free and Cued Selective Reminding Test

(*) At baseline: temporoparietal hypometabolism in FDG-PET or altered levels of amyloid-1-42 and p-tau in the CSF analysis

**Supplementary Table 2.** ADNI dataset diagnostic criteria.

|  | LMCI | EMCI | SMC | CN |
| --- | --- | --- | --- | --- |
| First visit | Subjective memory complaints | Subjective memory complaints | Subjective memory complaints | Free of memory complaints |
| Memory function (*) | Abnormal  Scores:   1. ≤8 for 16 or more years of education 2. ≤4 for 8-15 years of education 3. ≤2 for 0-7 years of education | Abnormal  Scores:   1. 9-11 for 16 or more years of education 2. 5-9 for 8-15 years of education 3. c. 3-6 for 0-7 years of education | Cognitive Change Index of zero.  Score within the normal range for cognition, and the informant does not equate the expressed concern with progressive memory impairment | Normal  Scores:   1. ≥9 for 16 or more years of education 2. ≥5 for 8-15 years of education 3. ≥3 for 0-7 years of education |
| MMSE | Between 24 and 30 (inclusive). | Between 24 and 30 (inclusive). | - | Between 24 and 30 (inclusive). |
| CDR | = 0.5  Memory Box ≥ 0.5 | = 0.5  Memory Box ≥ 0.5 | = 0 | = 0  Memory Box = 0 |
|  | General cognition and functional performance sufficiently preserved such that a diagnosis of AD cannot be made by the site physician at the time of the screening visit | General cognition and functional performance sufficiently preserved such that a diagnosis of AD cannot be made by the site physician at the time of the screening visit | - | Cognitively normal, based on an absence of significant impairment in cognitive functions or activities of daily living |

LMCI: Late Mild Cognitive Impairment, EMCI: Early Mild Cognitive Impairment, SMC: Subjective Memory Complaint; CN: Cognitively Normal, AD: Alzheimer’s disease

MMSE: Mini-Mental State Examination, CDR: Clinical Dementia Rating

(*) Documented by scoring within the education adjusted ranges on the Logical Memory II subscale (Delayed Paragraph Recall, Paragraph A only) from the Wechsler Memory Scale –Revised (the maximum score is 25).

####

#### 2. Results

**Clustering results: SI scores**

**Supplementary Table 3.** SI scores obtained for KMeans clustering with biomarkers data in the HCSC dataset.

| Clusters | Aβ(1-42) | tTau | pTau | Ratio | All Aβ(1-42) | All Ratio |
| --- | --- | --- | --- | --- | --- | --- |
| n = 2 | 0.6586 | 0.6546 | 0.6777 | 0.7448 | 0.4804 | 0.5291 |
| n = 3 | 0.5665 | 0.6156 | 0.6287 | 0.6370 | 0.4646 | **0.5527** |
| n = 4 | 0.5637 | 0.5702 | 0.5747 | 0.6055 | 0.4477 | 0.5279 |
| n = 5 | 0.5349 | 0.5713 | 0.5733 | 0.5756 | 0.4246 | 0.496 |
| n = 6 | 0.5483 | 0.5804 | 0.5801 | 0.5523 | 0.3873 | 0.4511 |
| n = 7 | 0.5660 | 0.5835 | 0.5706 | 0.5806 | 0.4024 | 0.3772 |
| n = 8 | 0.5659 | 0.5457 | 0.5695 | 0.5888 | 0.3815 | 0.373 |
| n = 9 | 0.5811 | 0.5552 | 0.5736 | 0.6021 | 0.3591 | 0.3706 |
| n = 10 | 0.5829 | 0.5542 | 0.5878 | 0.5784 | 0.3405 | 0.3735 |

All Aβ(1-42) and All Ratio: all biomarkers combined using Aβ(1-42) or Aβ(1-42)/Aβ(1-40) as amyloid representative, respectively. Bolded values represent the clustering solution evaluated in the main manuscript.

**Supplementary Table 4.** SI scores obtained for KMeans clustering with biomarkers data in the ADNI dataset.

| Clusters | Aβ(1-42) | tTau | pTau | All Aβ(1-42) |
| --- | --- | --- | --- | --- |
| n = 2 | 0.7071 | 0.6090 | 0.6306 | 0.4495 |
| n = 3 | 0.6662 | 0.5539 | 0.6003 | **0.4432** |
| n = 4 | 0.6563 | 0.5486 | 0.5454 | 0.4419 |
| n = 5 | 0.6739 | 0.5592 | 0.5487 | 0.4121 |
| n = 6 | 0.6673 | 0.5598 | 0.5458 | 0.4019 |
| n = 7 | 0.6647 | 0.5473 | 0.5316 | 0.4016 |
| n = 8 | 0.6640 | 0.5463 | 0.5407 | 0.407 |
| n = 9 | 0.6729 | 0.5606 | 0.5550 | 0.3861 |
| n = 10 | 0.6751 | 0.5563 | 0.5597 | 0.3767 |

All Aβ(1-42): all biomarkers combined using Aβ(1-42) as amyloid representative. Bolded values represent the clustering solution evaluated in the main manuscript.

**AT(N) system categories survival analysis**

**Supplementary Table 5.** Results obtained for survival Cox models comparing all AT(N) categories’ pairs present in the ADNI dataset.

| AT(N) categories pair | | Total | Distribution (True*) | | HR (CI) | p-value |
| --- | --- | --- | --- | --- | --- | --- |
| A-T-(N-) | A-T-(N+) | 169 | 14 | 0 | 0.00 | 1.00 |
|  | A+T-(N-) | 245 | 14 | 21 | 2.97 (1.50-5.88) | **<0.005** |
|  | A+T+(N-) | 171 | 14 | 7 | 7786.69 (27.82-2.18e+06) | **<0.005** |
|  | A+T+(N+) | 250 | 14 | 59 | 19.04 (10.28-35.27) | **<0.005** |
| A-T+(N-) | A-T-(N-) | 166 | 0 | 14 | 3.00e+06 | 1.00 |
|  | A-T-(N+) | 13 | 0 | 0 | NA | NA |
|  | A-T+(N+) | 67 | 0 | 11 | 1.02e+07 | 1.00 |
|  | A+T+(N-) | 15 | 0 | 7 | 4.32e+08 | 0.99 |
|  | A+T-(N-) | 89 | 0 | 21 | 9.89e+06 | 0.99 |
|  | A+T+(N+) | 94 | 0 | 59 | 8.03e+07 | 1.00 |
| A-T+(N+) | A-T-(N-) | 223 | 11 | 14 | 0.48 (0.21-1.07) | 0.07 |
|  | A-T-(N+) | 70 | 11 | 0 | 0.00 | 0.99 |
|  | A+T-(N-) | 146 | 11 | 21 | 1.44 (0.67-3.09) | 0.35 |
|  | A+T+(N-) | 72 | 11 | 7 | 8.21 (2.95-22.79) | **<0.005** |
|  | A+T+(N+) | 151 | 11 | 59 | 7.61 (3.89-14.86) | **<0.005** |
| A+T-(N-) | A-T-(N+) | 92 | 21 | 0 | 0.00 | 1.00 |
|  | A+T+(N-) | 94 | 21 | 7 | 8.17 (3.15-21.16) | **<0.005** |
|  | A+T+(N+) | 173 | 21 | 59 | 5.10 (3.04-8.55) | **<0.005** |
| A+T+(N-) | A-T-(N+) | 18 | 7 | 0 | 0.00 | 0.99 |
|  | A+T+(N+) | 99 | 7 | 59 | 1.19 (0.54-2.65) | 0.67 |
| A+T+N+ | A-T-(N+) | 97 | 59 | 0 | 0.00 | 0.99 |

HR: Hazard Ratio; CI: Confidence Interval

* Number of patients that have a dementia change in the first and second AT(N) categories compared

CIs are only showed if the Cox model yielded not-extreme HR values (e.g. 0 or a extremely high value)

**Handling of extreme Aβ(1-42) values in ADNI**

An apparent counterintuitive result is pTau and tTau values were higher in cluster 0 than in cluster 1 in ADNI, probably due to an artifact in the data. ADNI cohort showed many saturated points at the 1700 pg/mL value (**Figure 3b**). The equivalent scatterplot obtained for ADNI in the work by Hansson et al., 2018 ^1^, shows an "L" shape, similar to the one obtained in HCSC (**Figure 3a**). The cited work included Aβ(1-42) values greater than 1700 pg/mL using an estimation from extrapolated calibration curves ^1^. We did not have access to these extrapolated values; therefore Aβ(1-42) values originally listed in the database as ">1700" were replaced with 1700. This handling of extreme Aβ(1-42) values most likely caused KMeans to include several patients from cluster 0 in cluster 1, resulting in subsequent artefacted results. When we performed the clustering discarding subjects with ">1700" values, we obtained much more similar results to HCSC results, with an increase in the value of Tau biomarkers according to the cluster progression. Supplementary Table 6 summarizes these results.

1. [Hansson O, Seibyl J, Stomrud E, et al. CSF biomarkers of Alzheimer’s disease concord with amyloid-β PET and predict clinical progression: A study of fully automated immunoassays in BioFINDER and ADNI cohorts. Alzheimers Dement. 2018;14(11):1470–1481.](https://www.zotero.org/google-docs/?broken=lvtS7h)

**Supplementary Table 6.** Statistical description of clusters obtained with all CSF biomarkers combined in the **ADNI** dataset, deleting patients with Aβ(1-42) > 1700 pg/mL.

| Variable | Cluster 0 | Cluster 1 | Cluster 2 | Statistic | p-value |
| --- | --- | --- | --- | --- | --- |
| Number | 175 | 161 | 83 | *NA* | *NA* |
| Aβ(1-42) | 1292.68 ± 248.74 | 642.40 ± 185.81 | 755.46 ± 198.57 | 307.56 | < 0.001 |
| pTau | 16.79 ± 5.20 | 22.42 ± 7.17 | 46.96 ± 11.53 | 341.93 | < 0.001 |
| tTau | 190.96 ± 57.83 | 231.28 ± 67.00 | 456.22 ± 100.45 | 298.65 | < 0.001 |
| Sex, Female (%) | 44.85 | 37.93 | 50.85 | 0.03 | 0.9832 |
| Age (years) at baseline | 72.34 ± 6.42 | 73.52 ± 6.16 | 72.76 ± 8.02 | 0.99 | 0.1938 |
| Education years | 16.61 ± 2.43 | 16.14 ± 2.80 | 15.81 ± 2.87 | 2.12 | 0.3046 |
| MMSE at baseline | 28.77 ± 1.47 | 28.38 ± 1.52 | 27.51 ± 1.91 | 13.14 | < 0.001 |

For continuous variables, values represent mean ± standard deviation.

p-values computed for ANOVA for continuous variables and the chi-squared test for continuous ones.

**Clustering results: HCSC cohort using Aβ(1-42)**


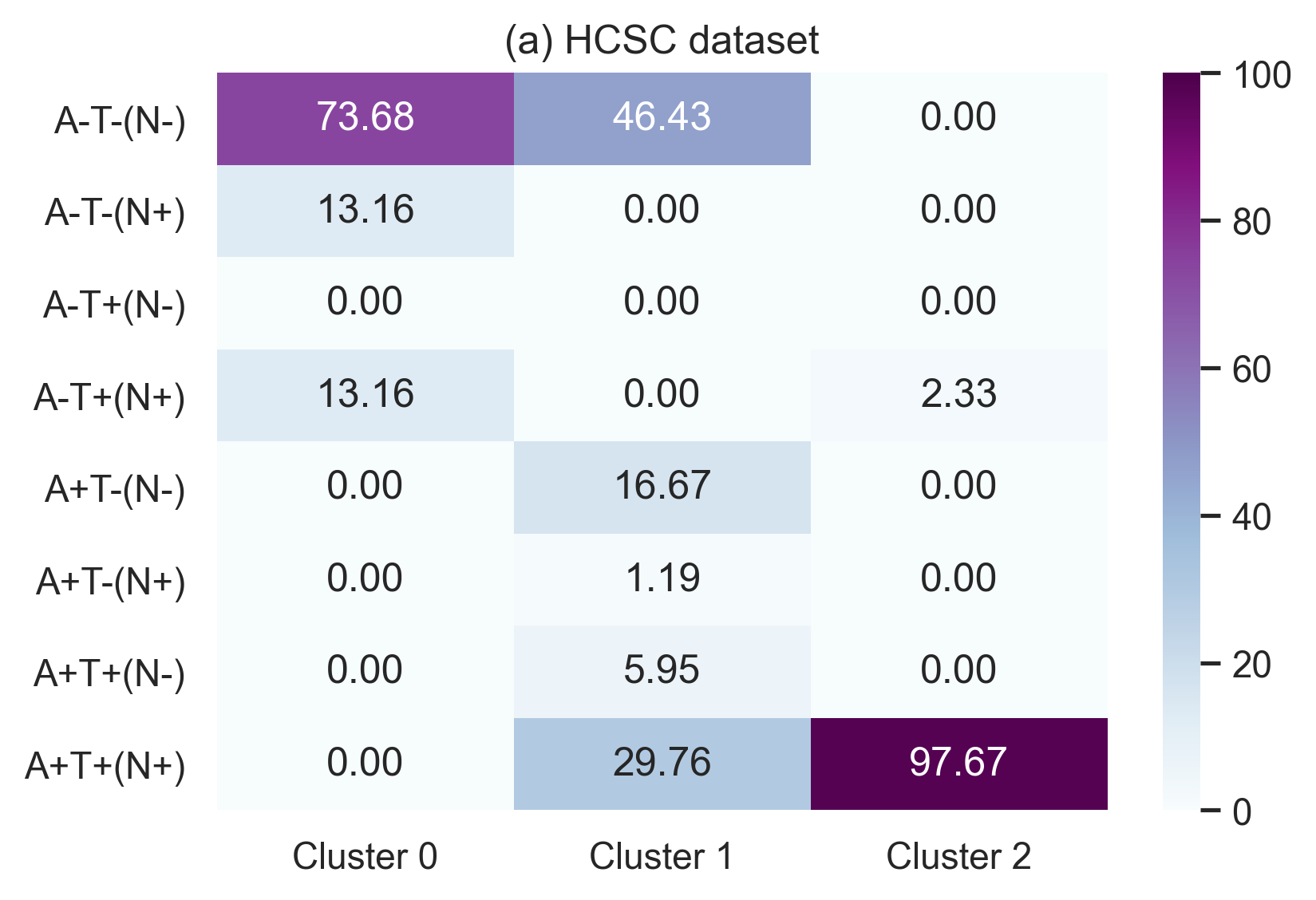


**Supplementary Figure 1.** Heatmap showing within-cluster distribution (%) of different AT(N) categories in HCSC dataset in clusters obtained with all biomarkers when using Aβ(1-42) value as the amyloid representative and not Aβ(1-42)/Aβ(1-40) as shown in Figure 3.
